# PD-1/PD-L1 inhibitors plus anti-angiogenic agents with or without chemotherapy versus PD-1/PD-L1 inhibitors plus chemotherapy as second or later-line treatment for patients with advanced non-small cell lung cancer: A real-world retrospective cohort study

**DOI:** 10.3389/fimmu.2022.1059995

**Published:** 2022-12-07

**Authors:** Shubin Chen, Haowen Wei, Wenhua Zhao, Wei Jiang, Ruiling Ning, Shaozhang Zhou, Liping Tan, Huilin Wang, Cuiyun Su, Jianbo He, Aiping Zeng, Yun Zhao, Qitao Yu

**Affiliations:** ^1^ Medical Oncology of Respiratory, Guangxi Cancer Hospital and Guangxi Medical University Affiliated Cancer Hospital, Nanning, China; ^2^ Department of Hepatobiliary Surgery, Key Laboratory of Early Prevention and Treatment for Regional High Frequency Tumor, Ministry of Education, Guangxi Cancer Hospital and Guangxi Medical University Affiliated Cancer Hospital, Nanning, China

**Keywords:** advanced non-small cell lung cancer, second or later-line therapy, PD-1/PD-L1 inhibitors, anti-angiogenic agents, real-world study

## Abstract

**Background:**

The aim of this study was to assessment the efficacy and safety of Programmed cell death protein 1 (PD-1)/Programmed cell death-Ligand protein 1 (PD-L1) inhibitors plus anti-angiogenic agents with or without chemotherapy versus PD-1/PD-L1 inhibitors plus chemotherapy as second or later-line treatment for patients with advanced non-small cell lung cancer.

**Methods:**

In this study, pre-treatment clinical and laboratory indicators from 73 patients with advanced non-small cell lung cancer were retrieved for retrospective analysis. According to the therapy regimes they received, the patients were separated into groups, PD-1/PD-L1 inhibitors plus chemotherapy group (PC group), PD-1/PD-L1 inhibitors plus anti-angiogenic agents’ group (PA group), PD-1/PD-L1 inhibitors plus anti-angiogenic agents plus chemotherapy group (PAC group). Cox’s proportional hazards regression model and Kaplan-Meier (KM) curves were used to assess the connection between treatment regimens and progression free survival (PFS) and overall survival (OS). In addition, the association of treatment regimens with the risk of disease progression and death was evaluated by subgroup analysis.

**Results:**

The average age of the enrolled patients was 58.2 ± 10.2 years and 75.3% were male. Multivariate analyses showed that patients in PA group (Disease progression: HR 0.4, P=0.005. Death: HR 0.4, P=0.024) and PAC group (Disease progression: HR 0.3, P=0.012. Death: HR 0.3, P=0.045) had a statistically significant lower hazard ratio (HR) for disease progression and death compared to patients in PC group. Kaplan-Meier analysis showed that patients in PA group (mPFS:7.5 vs.3.5, P=0.00052. mOS:33.1 vs.21.8, P=0.093) and PAC group (mPFS:5.1 vs.3.5, P=0.075. mOS:37.3 vs.21.8, P=0.14) had a longer PFS and OS compared to patients in PC group. In all the pre-defined subgroups, patients in PA and PAC groups showed a decreasing trend in the risk of disease progression and death in most subgroups. The patients in PA group (DCR:96.3% vs.58.3%, P=0.001) and PAC group (DCR:100% vs.58.3%, P=0.019) had a better disease control rate (DCR) than patients in PC group.

**Conclusion:**

PD-1/PD-L1 inhibitors plus anti-angiogenic agents with or without chemotherapy were superior to PD-1/PD-L1 inhibitors plus chemotherapy as second or later-line treatment in patients with advanced non-small cell lung cancer.

## Introduction

Lung cancer is one of the cancers that poses the greatest menace to people’s health and lives. According to the World Health Organization’s International Agency for Research on Cancer’s latest “Global Cancer Statistics 2020” data, lung cancer incidence and mortality rates in China were significantly higher, with men accounting for the highest incidence and mortality rates of all malignant tumors, and women accounting for the second highest incidence and first highest mortality rates (1).

85% of lung cancer are non-small cell lung cancer (NSCLC), which is the main type of lung cancer (2). Patients with stage I-III non-small cell lung cancer could be cured surgically, with a 5-year survival rate of approximately 70% (3). The 5-year survival rate for advanced non-small cell lung cancer is only 5% (4). Due to the lack of typical symptoms in lung cancer patients, about 62 percent of non-small cell lung cancer patients receive a stage IV diagnosis at their initial diagnosis (5). The most popular treatments for people with advanced non-small cell lung cancer include chemotherapy, targeted therapy, and immunotherapy (6). Although tyrosine kinase inhibitors (TKIs) have improved survival in patients with advanced non-small cell lung cancer who have a driver-gene, the 5-year survival rate for patients with driver-negative advanced non-small cell lung cancer remains poor due to TKIs treatment unsuitability. Exploring the effectiveness of various treatment methods is therefore urgently needed to assist physicians in perfecting their treatment plans.

The popularity of immune checkpoint inhibitors (ICIs) in the management of advanced non-small cell lung cancer has grown exponentially. Only those with high levels of PD-L1 expression could get PD-1/PD-L1 inhibitor monotherapy. Notwithstanding, combination regimens of PD-1/PD-L1 inhibitors with a variety of other medications (chemotherapy, anti-angiogenic drugs, and other immunotherapeutic agents) are increasingly being explored in immunotherapy clinical research because the benefit of PD-1/PD-L1 inhibitors monotherapy is constrained in this group of people with low or negative PD-L1 expression. KEYNOTE-189 and KEYNOTE-407 are two clinical trials that have produced promising results (7) (8), have discovered that combining PD-1/PD-L1 inhibitors with chemotherapy significantly improved progression-free survival and overall survival in patients with advanced non-small cell lung cancer who were driver-negative, lowering the risk of disease progression and death.

Small molecule inhibitors like anlotinib, apatinib, and lenvatinib, as well as monoclonal antibodies like bevacizumab are anti-angiogenic agents that reduce tumor angiogenesis by disrupting the VEGF signaling pathway, resulting in anti-tumor effects. Clinical trials, such as BEYOND (9) and ALTER0303 (10), have shown that this class of medicines is effective in the treatment of advanced non-small cell lung cancer. Several clinical trials have published results on whether immunotherapy combined with anti-angiogenic agents could be an effective treatment option. IMpower150 (11) showed that atezolizumab in combination with chemotherapy and bevacizumab was effective in extending progression-free survival and overall survival in patients with advanced non-small cell lung cancer. KEYNOTE-524 (12) showed an objective response rate (ORR) of 33.3% for pembrolizumab in combination with lenvatinib as the first-line treatment for advanced non-small cell lung cancer. These results provide an evidence-based basis for the treatment regimen of PD-1/PD-L1 inhibitors in combination with anti-angiogenic agents.

There are presently just a few second or later-line therapeutic options available for patients with advanced NSCLC. Some clinicians prefer PD-1/PD-L1 inhibitors plus chemotherapy or PD-1/PD-L1 inhibitors plus anti-angiogenic agents with or without chemotherapy, however real-world data on the efficacy of PD-1/PD-L1 inhibitors plus anti-angiogenic with or without chemotherapy therapies is currently lacking. The efficacy and safety of PD-1/PD-L1 inhibitors plus anti-angiogenic with or without chemotherapy versus PD-1/PD-L1 inhibitors plus chemotherapy as second or later-line therapy for advanced non-small cell lung cancer patients were further investigated in this study using real-world clinical data analysis.

## Materials and methods

### Study subjects and design

**Figure 1:**
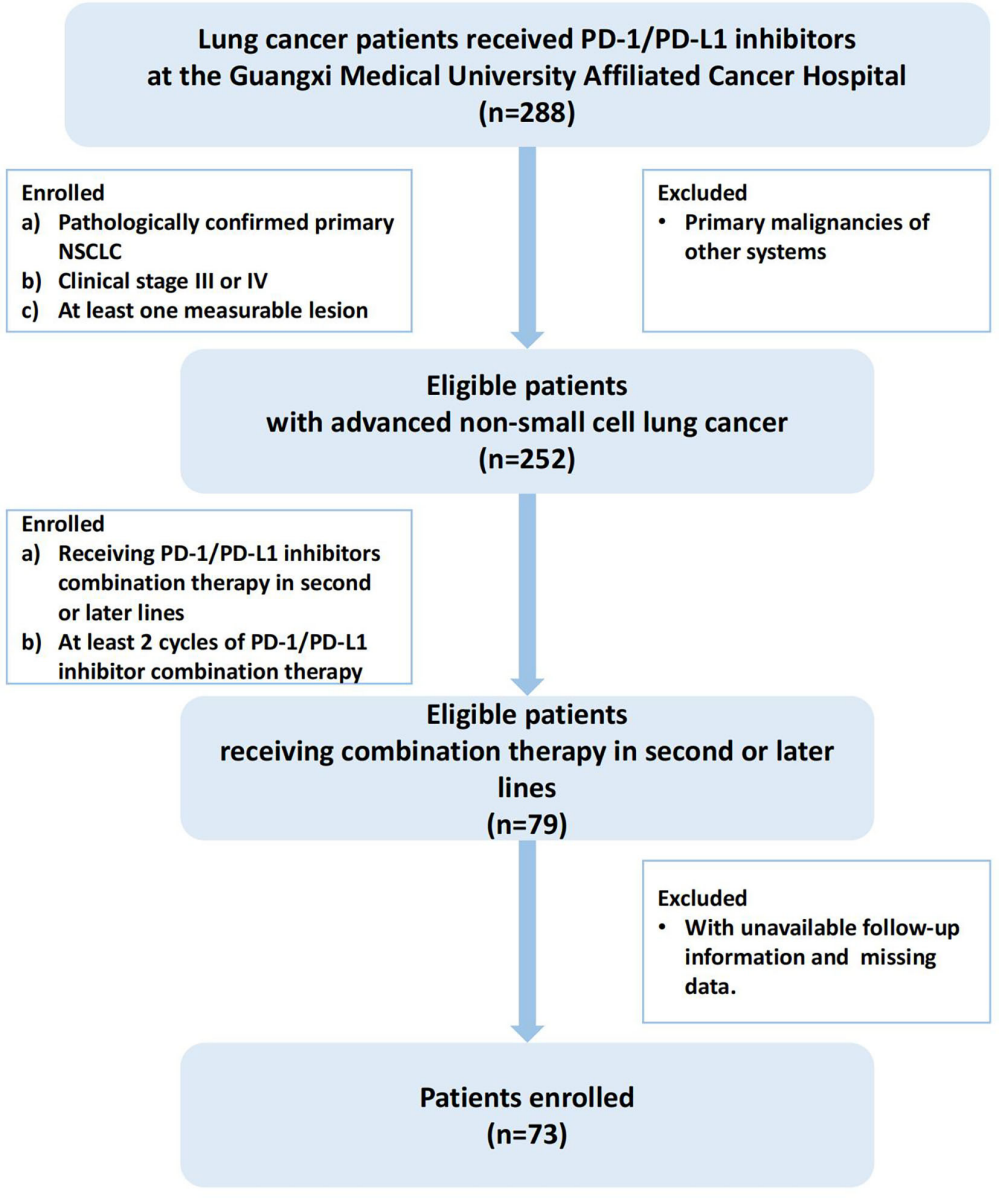
Flowchart of the study.

Lung cancer patients receiving PD-1/PD-L1 inhibitors at the Guangxi Cancer Hospital from January 1, 2018 to December 1, 2021 were included for further screening, and the screening criteria for the cohort study were as follows: (1) pathologically confirmed primary NSCLC (the 5th edition of the WHO Thoracic Tumor Classification); (2) clinical stage III(unresectable Stage IIIB and IIIC) or IV (the 8th edition of the TNM staging system); (3) exclude cases with primary malignancies in other systems; (4)at least one measurable lesion; (5) receiving PD-1/PD-L1 inhibitors combination therapy (chemotherapy, anti-angiogenic or both) in second or later lines; (6) receiving at least 2 cycles of PD-1/PD-L1 inhibitor combination therapy (21 days for 1 cycle); (7) exclude cases where follow-up information was not available and cases with missing data. A total of 73 patients met these criteria and were ultimately included in this cohort study, and the screening process and results are shown in **Figure 1**.

PD-1/PD-L1 inhibitors are frequently used in the first-line therapy of advanced NSCLC. Patients who were unable to utilize PD-1/PD-L1 inhibitors in the first-line for different reasons or patients whose EGFR-TKI treatment failed and selected PD-1/PD-L1 inhibitors as follow-up therapy made up a portion of the study’s patient population. Another part of the patients failed the first-line PD-1/PD-L1 inhibitors plus chemotherapy, and then used PD-1/PD-L1 inhibitors combination therapy again in the later line. All included patients were treated with PD-1/PD-L1 inhibitors in second or later-line therapy, PD-1 inhibitors included pembrolizumab, nivolumab, camrelizumab, tislelizumab, sintilimab, toripalimab. PD-L1 inhibitors included atezolizumab and durvalumab. Combination regimens with chemotherapeutic agents including pemetrexed, gemcitabine, paclitaxel analogues (docetaxel, paclitaxel, albumin paclitaxel, paclitaxel liposomes), platinum analogues (carboplatin, cisplatin). Anti-angiogenic agents included bevacizumab, anlotinib, apatinib. The patients all got PD-1/PD-L1 inhibitor combination treatment for more than 2 cycles. Based on the therapy plans they underwent, the patients were separated into groups: PD-1/PD-L1 inhibitors plus chemotherapy (PC group), PD-1/PD-L1 inhibitors plus anti-angiogenic medicines (PA group), and PD-1/PD-L1 inhibitors plus chemotherapy plus anti-angiogenic agents (PAC group).

### Data collection and assessment

The hospital database’s collection of medical records was searched for relevant information. Patients who had not visited the hospital in more than three months were contacted by phone to follow up and got the necessary information, such as the patient’s tumor recurrence and prognosis. Tumor lesions were evaluated both before and after treatment, and a CT scan was utilized to gauge how well the therapy responded. All clinical and laboratory indicators were extracted from the patient’s medical records. Clinical indicators included pathological type, sex, age, ECOG-PS, clinical stage, smoking history, metastases, line of treatment and treatment plan. Laboratory indicators included the EGFR mutation and PD-L1 tumor cell proportion score (TPS).

Response Evaluation Criteria in Solid Tumors (RECIST) version 1.1 was applied to classify effectiveness into four categories: complete response (CR), partial response (PR), stable disease (SD) and progressive disease (PD). Objective response rate (ORR): CR+PR; Disease control rate (DCR)=CR+PR+SD. Adverse events (AEs) were evaluated by the National Cancer Institute Common Terminology Criteria for Adverse Events (CTCAE) 5.0. Progression-free survival (PFS) was calculated from the date of initiation of treatment with the study protocol to the date of disease progression, or to the time of the last follow-up. Overall survival (OS) was the time from the patient’s first anti-tumor drug treatment to death or the last follow-up.

The Guangxi Medical University Affiliated Cancer Hospital ‘s ethical committee gave its approval for this study. All processes and information collection for this study followed the ethical standards of the Research Committee of the Guangxi Medical University Affiliated Cancer Hospital.

### Statistical analyses

All statistical analyses were performed using the statistical package R and EmpowerStats software. We used frequencies, percentages or ratios for categorical variables and means ± standard deviations (SD) for continuous variables. χ2 or Fisher’s exact test (for categorical variables) was used to test for differences between study protocol groups. Survival curves were plotted using the Kaplan-Meier method, and differences were compared using the log-rank test. Cox regression was used for both univariate and multivariate analyses. Hazard ratios (HR) and 95% confidence intervals (CI) were calculated using Cox’s proportional hazards regression model. In the multivariate analysis, we adjusted the potentially confounding covariates, the covariates included in the adjustment were screened using EmpowerStats statistics, the screening criteria was: introduction of covariates in the basic model or removal of covariates from the full model had >10% impact on the regression coefficient of the study protocol groups. Subgroup analyses were used to assess the association between treatment plans and the risk of disease progression and death in different subgroups. In all analyses, P<0.05 was statistically significant.

## Results

### Baseline characteristics

The clinical baseline data for the patients were shown in **Table 1** by the study protocol. The study had 73 patients in all, with a mean age of 58.2 ± 10.2 years and a gender ratio of 75.3% men. The majority of the pathological types were adenocarcinomas (65.8%). 65 patients had an ECOG-PS < 2, and 42 patients had a history of smoking. Ten individuals (13.7%) had a stage III diagnosis, while 63 (86.3%) had a stage IV diagnosis. 39 (53.4%) patients received second-line treatment and 34 (46.4%) received later-line treatment. Sixteen patients had EGFR mutation and 10 patients had PD-L1 TPS ≥ 1%. Age, sex, smoking history, ECOG-PS, pathological type, brain metastasis, liver metastasis, bone metastasis, lung metastasis, line of treatment, clinical stage, EGFR mutation and PD-L1 TPS in the study protocol groups were not statistically significantly different. However, more patients did not have adrenal metastasis (P=0.022).

**Table 1:** Baseline clinicopathological features.

	Total n = 73	PC group n =36	PA group n =27	PAC group n=10	P-value
Age	58.2 ± 10.2				0.177
<65	51 (69.9%)	26 (72.2%)	16 (69.3%)	9 (90.0%)	
≥65	22 (30.1%)	10 (27.8%)	11 (40.7%)	1 (10.0%)	
Sex					0.931
female	18 (24.7%)	9 (25.0%)	7 (25.9%)	2 (20.0%)	
male	55 (75.3%)	27 (75.0%)	20 (74.1%)	8 (80.0%)	
Smoking History					0.944
never	31 (42.5%)	16 (44.4%)	11 (40.7%)	4 (40.0%)	
ever	42 (57.5%)	20 (55.6%)	16 (59.3%)	6 (60.0%)	
ECOG-PS					0.995
<2	65 (89.0%)	32 (88.9%)	24 (88.9%)	9 (90.0%)	
≥2	8 (11.0%)	4 (11.1%)	3 (11.1%)	1 (10.0%)	
Pathological Type					0.282
adenocarcinoma	48 (65.8%)	21 (58.3%)	18 (66.7%)	9 (90.0%)	
squamous cell carcinoma	20 (27.4%)	13 (36.1%)	6 (22.2%)	1 (10.0%)	
others	5 (6.8%)	2 (5.60%)	3 (11.1%)	0 (0.00%)	
Brain Metastasis					0.843
no	54 (74.0%)	26 (72.2%)	21 (77.8%)	7 (70.0%)	
yes	19 (26.0%)	10 (27.8%)	6 (22.2%)	3 (30.0%)	
Liver Metastasis					0.502
no	57 (78.1%)	27 (75.0%)	23 (85.2%)	7 (70.0%)	
yes	16 (21.9%)	9 (25.0%)	4 (14.8%)	3 (30.0%)	
Bone Metastasis					0.262
no	51 (69.9%)	25 (69.4%)	21 (77.8%)	5 (50.0%)	
yes	22 (30.1%)	11 (30.6%)	6 (22.2%)	5 (50.0%)	
Lung Metastasis					0.712
no	48 (65.8%)	22 (61.1%)	19 (70.4%)	7 (70.0%)	
yes	25 (34.2%)	14 (38.9%)	8 (29.6%)	3 (30.0%)	
Adrenal Metastasis					0.022
no	59 (80.8%)	32 (88.9%)	22 (81.5%)	5 (50.0%)	
yes	14 (19.2%)	4 (11.1%)	5 (18.5%)	5 (50.0%)	
Line of treatment					0.746
2	39 (53.4%)	18 (50.0%)	16 (59.3%)	5 (50.0%)	
>2	34 (46.6%)	18 (50.0%)	11 (40.7%)	5 (50.0%)	
Clinical Stage					0.765
III	10 (13.7%)	6 (16.7%)	3 (11.1%)	1 (10.0%)	
IV	63 (86.3%)	30 (83.3%)	24 (88.9%)	9 (90.0%)	
EGFR mutation					0.461
negative	37 (50.7%)	17 (47.2%)	15 (55.6%)	5 (50.0%)	
positive	16 (21.9%)	8 (22.2%)	4 (14.8%)	4 (40.0%)	
unknown	20 (27.4%)	11 (30.6%)	8 (29.6%)	1 (10.0%)	
PD-L1 TPS					0.167
<1%	7 (9.60%)	3 (8.30%)	1 (3.70%)	3 (30.0%)	
≥1%	10 (13.7%)	6 (16.7%)	3 (11.1%)	1 (10.0%)	
unknown	56 (76.7%)	27 (75.0%)	23 (85.2%)	6 (60.0%)	

ECOG-PS, Eastern Cooperative Oncology Group Performance Status; EGFR, Epidermal Growth Factor Receptor. PD-L1 TPS, Programmed Cell Death-Ligand 1 Tumor cell Proportion Score.

PC group: PD-1/PD-L1 inhibitors plus Chemotherapy.

PA group: PD-1/PD-L1 inhibitors plus anti-angiogenic agents.

PAC group: PD-1/PD-L1 inhibitors plus anti-angiogenic agents plus Chemotherapy.

### Univariate analyses of the relationship between study protocol groups and the risk of disease progression and death

**Tables 2**, **3** demonstrated the results of the univariate analysis. In terms of disease progression, there was no statistical significance in age, sex, smoking history, ECOG-PS, pathological type, brain metastasis, liver metastasis, bone metastasis, lung metastasis, adrenal metastasis, clinical stage, line of treatment, EGFR mutation and PD-L1 TPS. In terms of study protocol groups, the risk of disease progression was reduced by 70% (95% CI=0.2-0.6, P<0.001) in PA group patients and 50% (95% CI=0.2-1.1, P=0.071) in PAC group patients compared to PC group patients.

**Table 2:** Univariate cox regression analysis of clinical indicators to predict risk of disease progression.

	N	HR	(95% CI)	p value
Age				
<65	51 (69.9%)	1		
≥65	22 (30.1%)	0.7	(0.4, 1.3)	0.247
Sex				
female	18 (24.7%)	1		
male	55 (75.3%)	1.1	(0.6, 2.1)	0.69
Smoking History				
never	31 (42.5%)	1		
ever	42 (57.5%)	0.7	(0.4, 1.2)	0.148
ECOG-PS				
<2	65 (89.0%)	1		
≥2	8 (11.0%)	0.9	(0.3, 2.2)	0.767
Pathological Type				
adenocarcinoma	48 (65.8%)	1		
squamous cell carcinoma	20 (27.4%)	0.8	(0.4, 1.4)	0.412
others	2 (5.60%)	1	(0.3, 2.8)	0.961
Brain Metastasis				
no	54 (74.0%)	1		
yes	19 (26.0%)	1.8	(1.0, 3.3)	0.045
Liver Metastasis				
no	57 (78.1%)	1		
yes	16 (21.9%)	1.5	(0.8, 2.8)	0.169
Bone Metastasis				
no	51 (69.9%)	1		
yes	22 (30.1%)	1.3	(0.7, 2.3)	0.364
Lung Metastasis				
no	48 (65.8%)	1		
yes	25 (34.2%)	1.3	(0.8, 2.3)	0.314
Adrenal Metastasis				
no	59 (80.8%)	1		
yes	14 (19.2%)	1.1	(0.6, 2.2)	0.774
Line of treatment				
2	39 (53.4%)	1		
>2	34 (46.6%)	1.2	(0.7, 2.1)	0.453
Stage				
III	10 (13.7%)	1		
IV	63 (86.3%)	1.5	(0.7, 3.6)	0.32
EGFR mutation				
negative	37 (50.7%)	1		
positive	16 (21.9%)	1.2	(0.7, 3.6)	0.523
unknown	20 (27.4%)	0.5	(0.3, 1.0)	0.061
PD-L1 TPS				
<1%	7 (9.60%)	1		
≥1%	10 (13.7%)	1.8	(0.7, 4.8)	0.229
unknown	56 (76.7%)	0.7	(0.3, 1.5)	0.362
Treatment				
PC group	36 (49.3%)	1		
PA group	27 (37.0%)	0.3	(0.2, 0.6)	<0.001
PAC group	10 (13.7%)	0.5	(0.2, 1.1)	0.071

HR, Hazard Ratio; CI, conﬁdence interval; ECOG-PS, Eastern Cooperative Oncology Group Performance Status; EGFR, Epidermal Growth Factor Receptor; PD-L1 TPS, Programmed Cell Death-Ligand 1 Tumor cell Proportion Score.

PC group: PD-1/PD-L1 inhibitors plus Chemotherapy.

PA group: PD-1/PD-L1 inhibitors plus anti-angiogenic agents.

PAC group: PD-1/PD-L1 inhibitors plus anti-angiogenic agents plus Chemotherapy.

**Table 3:** Univariate cox regression analysis of clinical indicators to predict risk of death.

	N	HR	(95% CI)	p value
Age				
<65	51 (69.9%)	1		
≥65	22 (30.1%)	0.6	(0.3, 1.3)	0.221
Sex				
female	18 (24.7%)	1		
male	55 (75.3%)	1.2	(0.6, 2.3)	0.647
Smoking History				
never	31 (42.5%)	1		
ever	42 (57.5%)	0.8	(0.5, 1.5)	0.516
ECOG-PS				
<2	65 (89.0%)	1		
≥2	8 (11.0%)	0.8	(0.3, 2.0)	0.666
Pathological Type				
adenocarcinoma	48 (65.8%)	1		
squamous cell carcinoma	20 (27.4%)	1.2	(0.6, 2.3)	0.688
others	2 (5.60%)	0.8	(0.3, 2.4)	0.732
Brain Metastasis				
no	54 (74.0%)	1		
yes	19 (26.0%)	0.5	(0.2, 1.0)	0.051
Liver Metastasis				
no	57 (78.1%)	1		
yes	16 (21.9%)	1.1	(0.5, 2.1)	0.817
Bone Metastasis				
no	51 (69.9%)	1		
yes	22 (30.1%)	0.9	(0.5, 1.7)	0.855
Lung Metastasis				
no	48 (65.8%)	1		
yes	25 (34.2%)	1.2	(0.6, 2.2)	0.583
Adrenal Metastasis				
no	59 (80.8%)	1		
yes	14 (19.2%)	1	(0.4, 2.1)	0.932
Line of treatment				
2	39 (53.4%)	1		
>2	34 (46.6%)	0.4	(0.2, 0.7)	0.002
Stage				
III	10 (13.7%)	1		
IV	63 (86.3%)	0.6	(0.2, 1.7)	0.349
EGFR mutation				
negative	37 (50.7%)	1		
positive	16 (21.9%)	0.5	(0.3, 1.1)	0.523
unknown	20 (27.4%)	0.7	(0.3, 1.4)	0.286
PD-L1 TPS				
<1%	7 (9.60%)	1		
≥1%	10 (13.7%)	1	(0.3, 3.0)	0.969
unknown	56 (76.7%)	0.4	(0.2, 1.1)	0.067
Treatment				
PC group	36 (49.3%)	1		
PA group	27 (37.0%)	0.6	(0.3, 1.1)	0.1
PAC group	10 (13.7%)	0.4	(0.2, 1.3)	0.126

HR, Hazard Ratio; CI, conﬁdence interval; ECOG-PS, Eastern Cooperative Oncology Group Performance Status; EGFR, Epidermal Growth Factor Receptor; PD-L1 TPS, Programmed Cell Death-Ligand 1 Tumor cell Proportion Score.

PC group: PD-1/PD-L1 inhibitors plus Chemotherapy.

PA group: PD-1/PD-L1 inhibitors plus anti-angiogenic agents.

PAC group: PD-1/PD-L1 inhibitors plus anti-angiogenic agents plus Chemotherapy.

Patients receiving later-line treatment had a statistically significant 60% decreased risk of death than those receiving second-line treatment (P=0.002). There was no statistical significance for the remaining clinical markers. For the study protocol, patients in the PA group had a 40% (95% CI=0.3-1.1, P=0.100) lower likelihood of dying than those in the PC group, while those in the PAC group had a 60% (95% CI=0.2-1.3, P=0.126) lower risk.

### Multivariate Cox regression analyses after adjusting the potentially confounding covariates

To avoid interaction of clinical characteristics parameters, multivariate Cox regression analysis was used to determine the independent predictability of PA group and PAC group in terms of disease progression and death (**Tables 4**, **5**).

**Table 4:** Unadjusted and adjusted cox proportional hazards model for disease progression.

	N	Unadjusted HR (95% CI)	P-value	Fully Adjusted HR (95% CI)	P-value
Treatment					
PC group	36 (49.3%)	1.0		1.0	
PA group	27 (37.0%)	0.3 (0.2, 0.6)	<0.001	0.4 (0.2, 0.7)	0.005
PAC group	10 (13.7%)	0.5 (0.2, 1.1)	0.071	0.3 (0.1, 0.8)	0.012

Fully adjusted model adjusts for Smoking history; Pathological Type; Brain Metastasis; Bone Metastasis; Adrenal Metastasis; EGFR mutation; PD-L1 TPS.

PC group: PD-1/PD-L1 inhibitors plus Chemotherapy.

PA group: PD-1/PD-L1 inhibitors plus anti-angiogenic agents.

PAC group: PD-1/PD-L1 inhibitors plus anti-angiogenic agents plus Chemotherapy.

HR, Hazard Ratio; CI, conﬁdence interval; ECOG-PS, Eastern Cooperative Oncology Group Performance Status; EGFR, Epidermal Growth Factor Receptor. PD-L1 TPS, Programmed Cell Death-Ligand 1 Tumor cell Proportion Score.

**Table 5:** Unadjusted and adjusted cox proportional hazards model for death.

	N	Unadjusted HR (95% CI)	P-value	Fully Adjusted HR (95% CI)	P-value
Treatment					
PC group	36 (49.3%)	1.0		1.0	
PA group	27 (37.0%)	0.6 (0.3, 1.1)	0.1	0.4 (0.2, 0.9)	0.024
PAC group	10 (13.7%)	0.4 (0.2, 1.3)	0.126	0.3 (0.1, 1.0)	0.045

Fully adjusted model adjusts for Age; ECOG-PS; Brain Metastasis; Adrenal Metastasis; Line of therapy; EGFR mutation; PD-L1 TPS.

PC group: PD-1/PD-L1 inhibitors plus Chemotherapy.

PA group: PD-1/PD-L1 inhibitors plus anti-angiogenic agents.

PAC group: PD-1/PD-L1 inhibitors plus anti-angiogenic agents plus Chemotherapy.

HR, Hazard Ratio; CI, conﬁdence interval; ECOG-PS, Eastern Cooperative Oncology Group Performance Status; EGFR, Epidermal Growth Factor Receptor. PD-L1 TPS, Programmed Cell Death-Ligand 1 Tumor cell Proportion Score.

Based on the results of the multivariate Cox’s proportional hazards regression model, we could see that PA group (Disease progression: HR 0.4, P=0.005. Death: HR 0.4, P=0.024) and PAC group (Disease progression: HR 0.3, P=0.012. Death: HR 0.3, P=0.045) had a statistically significant lower hazard ratio (HR) for disease progression and death compared to patients in PC group.

### Kaplan–Meier analyses

**Figure 2:**
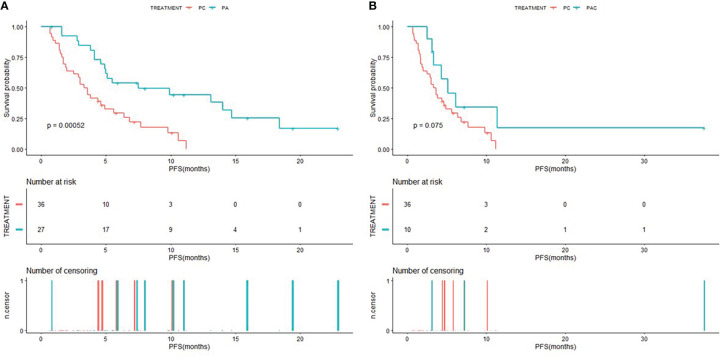
Kaplan-Meier (KM) curves of PFS **(A)** of patients in PC group versus PA group and PFS **(B)** of patients in PC group versus PAC group.

**Figure 3:**
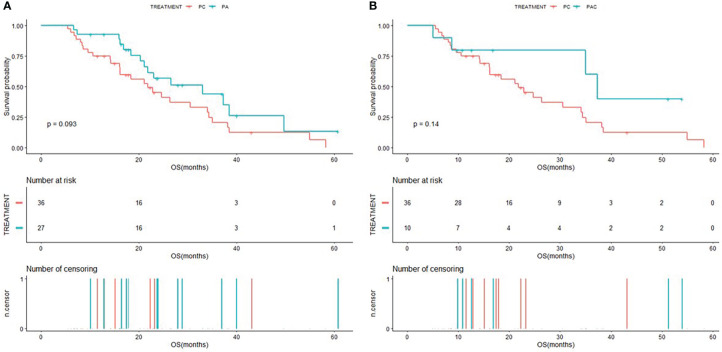
Kaplan-Meier (KM) curves of OS **(A)** of patients in PC group versus PA group and OS **(B)** of patients in PC group versus PAC group.

The Kaplan-Meier curves of PFS for patients in PC group compared to patients in PA group and PAC group were shown in **Figure 2**, and the Kaplan-Meier curves of OS for patients in PC group compared to patients in PA group and PAC group were shown in **Figure 3**. Kaplan-Meier analysis showed a median PFS of 3.5 months (95% CI=2.0-5.6) for patients in PC group and 7.5 months (95% CI=4.9-NA) for patients in PA group. 5.1 months (95% CI=3.3-NA) for patients in PAC group. The difference in PFS between patients in PC group and patients in PA group was significant (P=0.00052). In terms of OS, the median OS was 21.8 months (95% CI=16.2-34.4) for patients in PC group, 33.1 months (95% CI=21.1-NA) for patients in PA group and 37.3 months (95% CI=35.0-NA) for patients in PAC group.

### Stratified analyses

**Figure 4:**
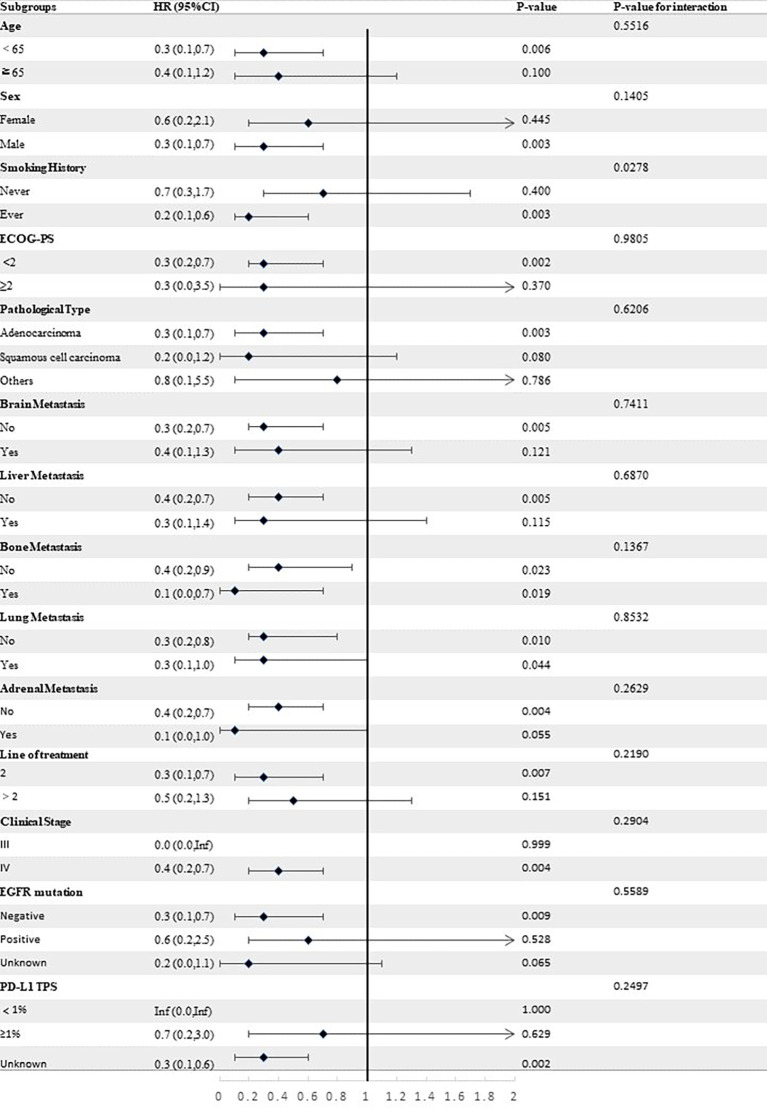
Forest plot of risk of disease progression for patients in PA group compared to PC group in different subgroups.

**Figure 5:**
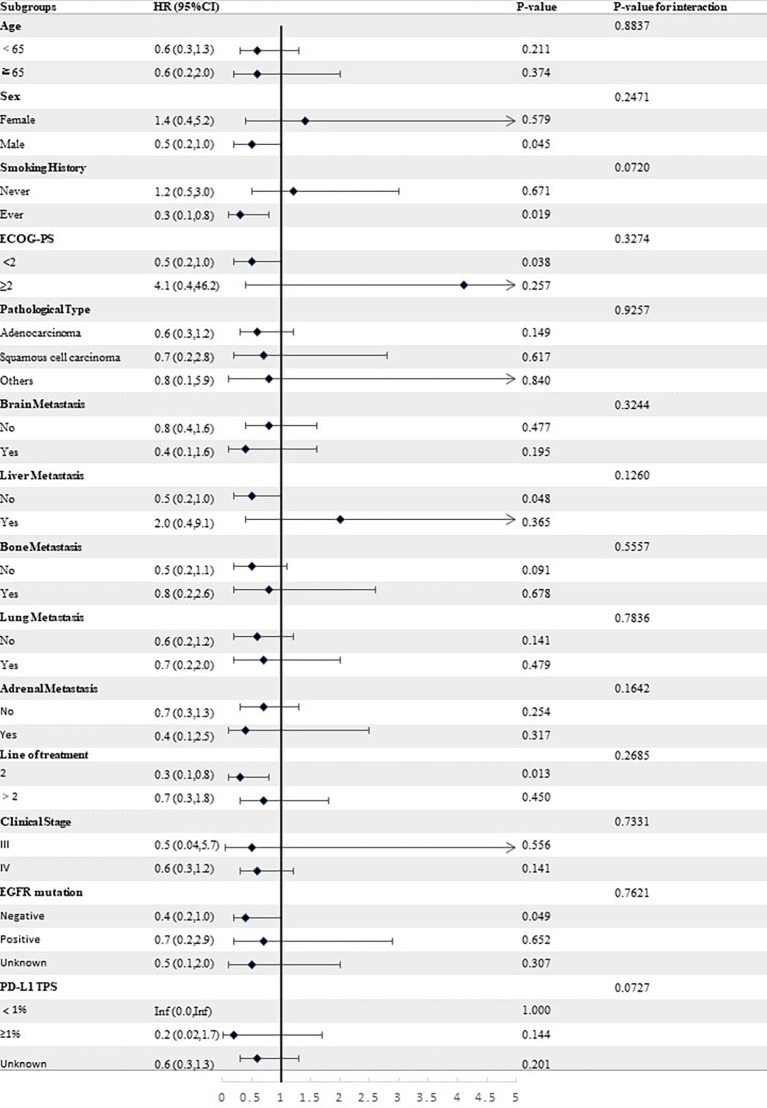
Forest plot of risk of disease progression for patients in PAC group compared to PC group in different subgroups.

**Figure 6:**
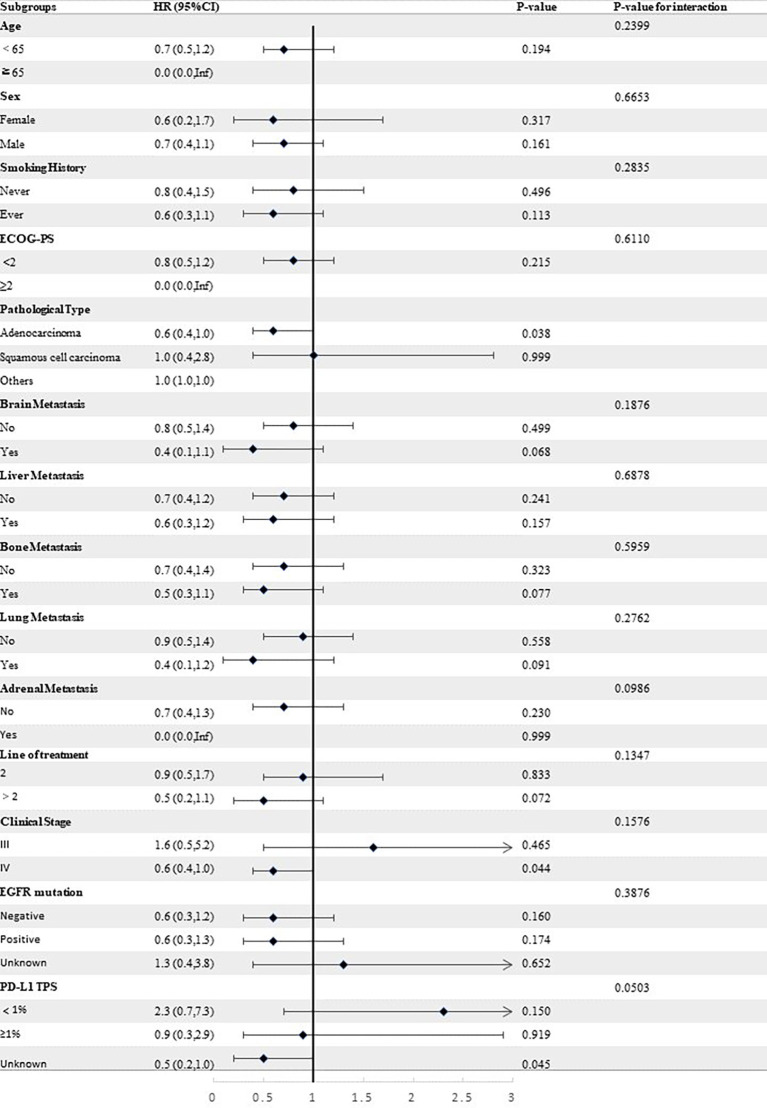
Forest plot of risk of death for patients in PA group compared to PC group in different subgroups.

**Figure 7:**
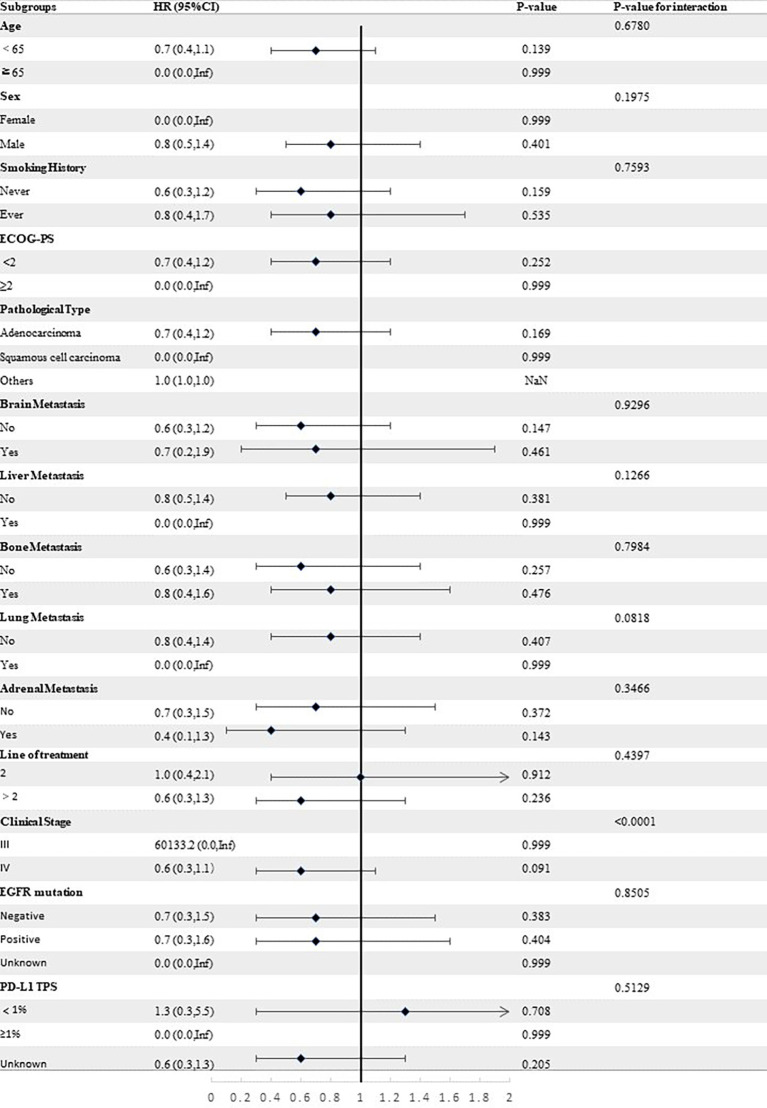
Forest plot of risk of death for patients in PAC group compared to PC group in different subgroups.

Stratified analyses were conducted to observe subgroup effect size trends for the study. The results of the stratified analyses of the risk of disease progression (**Figures 4**, **5**) and death (**Figures 6**, **7**) for patients in PA group and PAC group compared to those in PC group were represented by forest plots. Based on the results of the stratified analyses, it can be seen that patients in PA group and PAC group had a significantly lower risk of disease progression in most subgroups compared to patients in PC group, the interaction between the groups was not statistically significant. In terms of risk of death, patients in PA group and PAC group also had a significantly lower risk of death in most subgroups compared to patients in PC group. Moreover, it is worth noting that the risk of death was significantly higher for patients diagnosed with stage III in PAC group compared to those in PC group. However, the risk of death was reduced for patients diagnosed with stage IV in PAC group compared to those in PC group, with a P value of <0.0001 for the interaction analysis, which means that the prognosis may be different for patients in PAC group with different stage

### Confirmed objective response

The result of the confirmed objective response rates in the three groups were displayed in **Table 6**. None of the patients in the three groups were able to achieve complete response. The objective response rate (ORR) for the three groups was 13.9% in PC group, 11.1% in PA group and 20% in PAC group. The patients in PA group (DCR:96.3% vs.58.3%, P=0.001) and PAC group (DCR:100% vs.58.3%, P=0.019) had a better disease control rate (DCR) than patients in PC group.

**Table 6:** Summary of confirmed response assessed by RECIST version 1.1.

Confirmed Response	PC group	PA group	PAC group
**Best response**			
** Complete response (CR)**	0 (0%)	0 (0%)	0 (0%)
** Partial response(PR)**	5(13.9%)	3(11.1%)	2(20%)
** Stable disease(SD)**	16(44.4%)	23(85.2%)	8(80%)
** Progressive disease (PD)**	14(38.9%)	1(3.7%)	0(0%)
** Not evaluable**	1(2.8%)	0(%)	0(0%)
**Objective response rate(ORR)**	13.90%	11.10%	20%
P-value	0.802		
**Disease control rate (DCR)**	58.30%	96.30%	100%
P-value	<0.001		
PC vs PA	P=0.001		
PC vs PAC	P=0.019		

RECIST: Response Evaluation Criteria In Solid Tumors.

Objective response rate(ORR)= Complete response (CR)+ Partial response(PR).

Disease control rate (DCR)= Complete response (CR)+ Partial response(PR)+ Stable disease(SD).

Not evaluable= Patients who did not have 1 postbaseline imaging assessment.

PC group: PD-1/PD-L1 inhibitors plus Chemotherapy.

PA group: PD-1/PD-L1 inhibitors plus anti-angiogenic agents.

PAC group: PD-1/PD-L1 inhibitors plus anti-angiogenic agents plus Chemotherapy.

### Toxicities analyses

**Table 7** showed the incidence of treatment-related adverse events in the study protocol, with blood toxicity (27.4%) being the most common adverse event and abnormal renal function (1.4%) and pneumonia (1.4%) occurring relatively infrequently. Patients in PA group (18.5% vs.0%, P=0.011) and PAC group (30% vs.0%, P=0.008) had a greater proportion of hypertension than patients in PC group.

**Table 7:** Incidence of adverse events (AEs).

Event	PC group	PA group	PAC group	Total	P-value
**Blood toxicity**	12(33.3%)	6(22.2%)	2 (20%)	20(27.4%)	0.586
**Abnormal liver function**	2(5.6%)	2(7.4%)	0(0%)	4(5.5%)	1.000
**Abnormal renal function**	0(0%)	1(3.7%)	0(0%)	1(1.4%)	0.500
**Rash**	2(5.6%)	1(3.7%)	1(10%)	4(5.5%)	0.628
**Hypertension**	0(0%)	5(18.5%)	3(30%)	8(11%)	0.003*
**Abnormal thyroid function**	2(5.6%)	4(14.8%)	3(30%)	9(12.3%)	0.085
**Cardiotoxicity**	8(22.2%)	2(7.4%)	0(0%)	10(13.7%)	0.146
**Pneumonia**	1(2.8%)	0(0.0%)	0(0%)	1(1.4%)	1.000

PC group: PD-1/PD-L1 inhibitors plus Chemotherapy.

PA group: PD-1/PD-L1 inhibitors plus anti-angiogenic agents.

PAC group: PD-1/PD-L1 inhibitors plus anti-angiogenic agents plus Chemotherapy.

*PC vs PA P-value=0.011; PC vs PAC P-value=0.008.

## Discussion

The management of lung cancer has advanced quickly in this era of diverse medicines. However, there is still a bottleneck in the availability of second or later-line therapy for advanced non-small cell lung cancer. The TAX317 study (13) results showed that docetaxel used in second-line treatment of driver-negative advanced NSCLC significantly increased overall survival when compared to best supportive care (7 months vs 4.6 months), and the TAX317/TAX320 (14) studies established docetaxel as the standard chemotherapy regimen for second-line treatment of NSCLC. The paradigm of second or later-line therapy for patients with advanced NSCLC has been further altered by the development of immunotherapeutic drugs. The KEYNOTE-010 research (15) demonstrated that pembrolizumab resulted in longer OS than docetaxel in patients with PD-L1 TPS≥1%. The outcomes of CheckMate 078 (16) demonstrated that the effectiveness and safety performance of nivolumab in Chinese patients was similar to the worldwide trials CheckMate 017 and 057 (17) (18), with a median overall survival of 11.9 months in the nivolumab group compared to 9 months in the docetaxel group. When compared to docetaxel, nivolumab dramatically increased patient survival, lowering the chance of mortality by 36%. In the meanwhile, patients with PD-L1 TPS≥1% in OAK research (19), a phase 3 clinical research with atezolizumab, had a median OS of 15.7 months compared to 10.3 months when compared to docetaxel. In the ORIENT-3 study (20), the median PFS was also much longer in the sintilimab group, coming in at 4.30 months compared to 2.79 months in the docetaxel group. The RATIONALE303 study (21) was designed to evaluate the efficacy and safety of tislelizumab versus docetaxel in second or later-line treatment of patients with advanced NSCLC, showing a median PFS of 4.1 months vs. 2.6 months and a PFS rate of 23.3% vs. 5.7% at 12 months. The ALTER0303 study (10) demonstrated a median OS extension of 3.3 months for patients in the anlotinib arm compared to the placebo arm (9.6 months vs 6.3 months); and a median PFS extension of 4.0 months (5.4 months vs 1.4 months). Based on the results of the above, chemotherapy, PD-1/PD-L1 inhibitors or anti-angiogenic agents monotherapy have been approved by the Food and Drug Administration (FDA) and the National Medical Products Administration (NMPA) of China for the second or later-line treatment of patients with driver-negative advanced non-small cell lung cancer. Due to the paucity of scientific evidence supporting the use of combination regimens in the second or later-line treatment of patients with advanced non-small cell lung cancer, a number of clinical studies are being conducted worldwide to further examine the viability of combination regimens.

In this retrospective study, we fully evaluated the efficacy and safety of PD-1/PD-L1 inhibitors plus anti-angiogenic agents with or without chemotherapy in the second or later-line treatment of patients with advanced non-small cell lung cancer. The results showed that PD-1/PD-L1 inhibitors plus anti-angiogenic agents with or without chemotherapy were superior to PD-1/PD-L1 inhibitors plus chemotherapy in terms of progression-free survival in second or later-line treatment, and that two different combination regimens (PD-1/PD-L1 inhibitors plus anti-angiogenic agents and PD-1/PD-L1 inhibitors plus anti-angiogenic agents plus chemotherapy) reduced the risk of disease progression by 60% and 70%, respectively, compared to the PD-1/PD-L1 inhibitors plus chemotherapy, with consistent trends in results in subgroup analyses, and a better median PFS than the PD-1/PD-L1 inhibitors plus chemotherapy. In terms of overall survival, the multivariate Cox regression analysis showed a significantly lower risk of death with the two different combination regimens compared to the PD-1/PD-L1 inhibitors plus chemotherapy regimen, with statistically significant. In terms of Kaplan-Meier survival curves, patients in the two different combination regimens survived significantly better than those in the PD-1/PD-L1 inhibitors plus chemotherapy regimen. The overall survival data from this single center, small sample retrospective study should be further investigated in a multi-center study with a larger sample size because they may be statistically biased. Additionally, data on the overall survival of PD-1/PD-L1 inhibitors combined with anti-angiogenic agents, with or without chemotherapy regimens, warrant further investigation. Patients receiving one of the two alternative combination regimens had a considerably higher DCR than those receiving chemotherapy plus PD-1/PD-L1 inhibitors in terms of effectiveness. Although there was a greater prevalence of hypertension in the two distinct combination regimens, the majority of adverse events were grade 1-2, indicating the safety and tolerability of this treatment method. According to the findings of our retrospective study, patients who received PD-1/PD-L1 inhibitors plus anti-angiogenic agents with or without chemotherapy had numerically better OS and PFS than participants in the previous KEYNOTE-010, OAK, CheckMate 078, ORIENT-3, and RATIONALE303 studies. This proves the reliability of the data from our retrospective study.

Additionally, several academics have offered convincing explanations for the processes at play when PD-1/PD-L1 inhibitors are combined with other agents. Chemotherapy slows the development of tumors mostly by halting the cell cycle, preventing DNA replication, upsetting cellular metabolism, or blocking microtubule assembly (22). Through increased production of 2-microglobulin and changes to the peptide antigen repertoire expressed on HLA class I, gemcitabine can considerably upregulate the expression of human leukocyte antigens (HLA)-A, B, and C. Topotecan, which increases HLA class I expression by activating the NF-B/Interferon/MHC-I signaling axis, exhibits a similar behavior (23). Oxaliplatin and anthracycline are two examples of cytotoxic chemotherapy agents that can cause immunogenic cell death and activate the body’s natural defenses against tumors (24). Additionally, through enhancing mitochondrial biogenesis, pemetrexed increase the activation of tumor-infiltrating lymphocytes (TILs) (25). In animal models, it has been shown that chemotherapy and PD-1/PD-L1 inhibitors work in concert (26).

The tumor microenvironment (TME), a dynamic ecosystem, is made up of a variety of soluble chemicals, fibroblasts, stromal cells, blood vessels, tumor cells, and immune cells (27). Regulatory T cells (Tregs), myeloid-derived suppressor cells (MDSCs), tumor-associated macrophages (TAMs), and immature dendritic cells (ImDCs) are among the numerous immunological suppressor cells encountered in the TME (28). The abnormal morphology of tumor vascular endothelial cells and the loose connections between endothelial cells and different basement membranes ultimately lead to a heterogeneous blood perfusion of tumor cells and a hypoxia and acidosis microenvironment (29). Hypoxia further promotes infiltration of these suppressive immune cells by inducing the expression of chemokines that recruit suppressive immune cells. For example, in the presence of C-C Motif Chemokine Ligand 22 (CCL22) and C-C Motif Chemokine Ligand 28 (CCL28), Tregs are further promoted into the tumor cells (30). Vascular Endothelial Growth Factors (VEGFs), which include VEGF-A, VEGF-B, VEGF-C, VEGF-D, VEGF-E, VEGF-F and Placental Growth Factor (PIGF), are a group of secreted glycoproteins that are crucial for the angiogenesis of TME (31). VEGFR-1, 2 and 3 are the three VEGF receptors, and the pro-angiogenic effect of VEGF is primarily mediated by the binding of VEGF-A and VEGFR-2 receptors. The VEGF/VEGFR signaling pathway inhibits anti-tumor immune responses not only by inducing a hypoxic microenvironment, but also through other complex mechanisms to produce immunosuppressive effects.

Dendritic Cells (DCs) are specialized antigen-presenting Cells (APCs) that play a key role in the antitumor immune response. In the presence of tumor antigens, DCs migrate and become mature during the migration process. Mature DCs activate T cells to exert their anti-tumor effects (32). Immature DCs (ImDCs), because of the absence of co-stimulatory molecules, result in the inability of T cells to activate properly. According to a publication, VEGF inhibits DC maturation by binding to VEGFR-2 on their surface and activating the NF-kB signaling pathway (33). The maturation and differentiation of DCs were hampered by high amounts of VEGF in a mouse model, which provided additional confirmation of this conclusion (34).

Myeloid-derived Suppressor Cells (MDSCs) conduct a variety of mechanisms to suppress anti-tumor immune responses, such as depleting lymphocytes of nutrients, reducing the viability of transit lymphocytes, generating oxidative stress and inducing Tregs to differentiate (35). It has been shown that VEGF causes an increase in MDSCs and suppresses anti-tumor immune responses because VEGF expression over-activates Janus Kinases 2/Signal Transducer and Activator of Transcription 3 (Jak2/STAT3) signaling, leading to aberrant myeloid differentiation in tumors (36). In a mouse tumor model, the concentration of intratumoral MDSCs correlated with the concentration of VEGF, and the infusion of VEGF into tumor-free normal mice significantly increased the level of MDSCs in mice (37).

Tumor-associated macrophages (TAMs) come in two varieties: M1 and M2. A number of pro-inflammatory substances, immune activators, and chemokines are released by M1 TAMs, and these substances have anti-tumor effects through cytophagocytosis, acute pro-inflammatory reactions, and immune activation responses. By secreting immunosuppressive factors, cytokines and growth factors, M2 TAMs inhibit the proliferation and activation of T cells, regulate and promote the Th2 immune response, promote tumor cell growth, participate in tumor angiogenesis and promote tumor infiltration and metastasis (38). VEGF signaling promotes a step-change in TAMs from the M1 to the M2 phenotype, in addition to recruiting TAMs into tumors (39).

Regulatory T Cells (Tregs) inhibit the action of T cells on tumors (40). Tregs are activated and their immunosuppressive function is enhanced by Neurofibrillin-1 mediation, and VEGF binds directly to Neurofibrillin-1 and induces Tregs to migrate into the tumor (41). It has also been shown that VEGF expression is positively correlated with the level of Tregs in tumors and that VEGFR-2 is more abundant in Tregs compared to other T cells (42, 43), suggesting an important role for VEGF signaling in the activation and induction of Tregs. VEGF further exerts immunosuppressive effects by affecting Tregs.

T-cells may directly destroy tumor cells, which causes an immune response that is anti-tumor. The finding that VEGF was found to have a low expression in tumor-derived T cells (44) was validated, pointing to a relationship between VEGF and T cells. Further research indicated that the severe thymic atrophy caused by VEGF-A infusion in a tumor-bearing mouse model was caused by a significant decrease in CD4+/CD8+ thymocytes (45). More research was done on this phenomenon, and it was shown that co-repressor molecule production in CD8+ T cells enhances T cell depletion, which is also boosted by VEGF-A (46). VEGF not only affects the antitumor immune response by influencing the activation of T cells, but also reduces the expression of adhesion molecules on immune cells and endothelial cells that are essential for T cell infiltration, such as Vascular Cell Adhesion Molecule-1 (VCAM-1) and Intercellular Cell Adhesion Molecule-1 (ICAM-1) (47), thereby further affecting the immune response.

**Figure 8:**
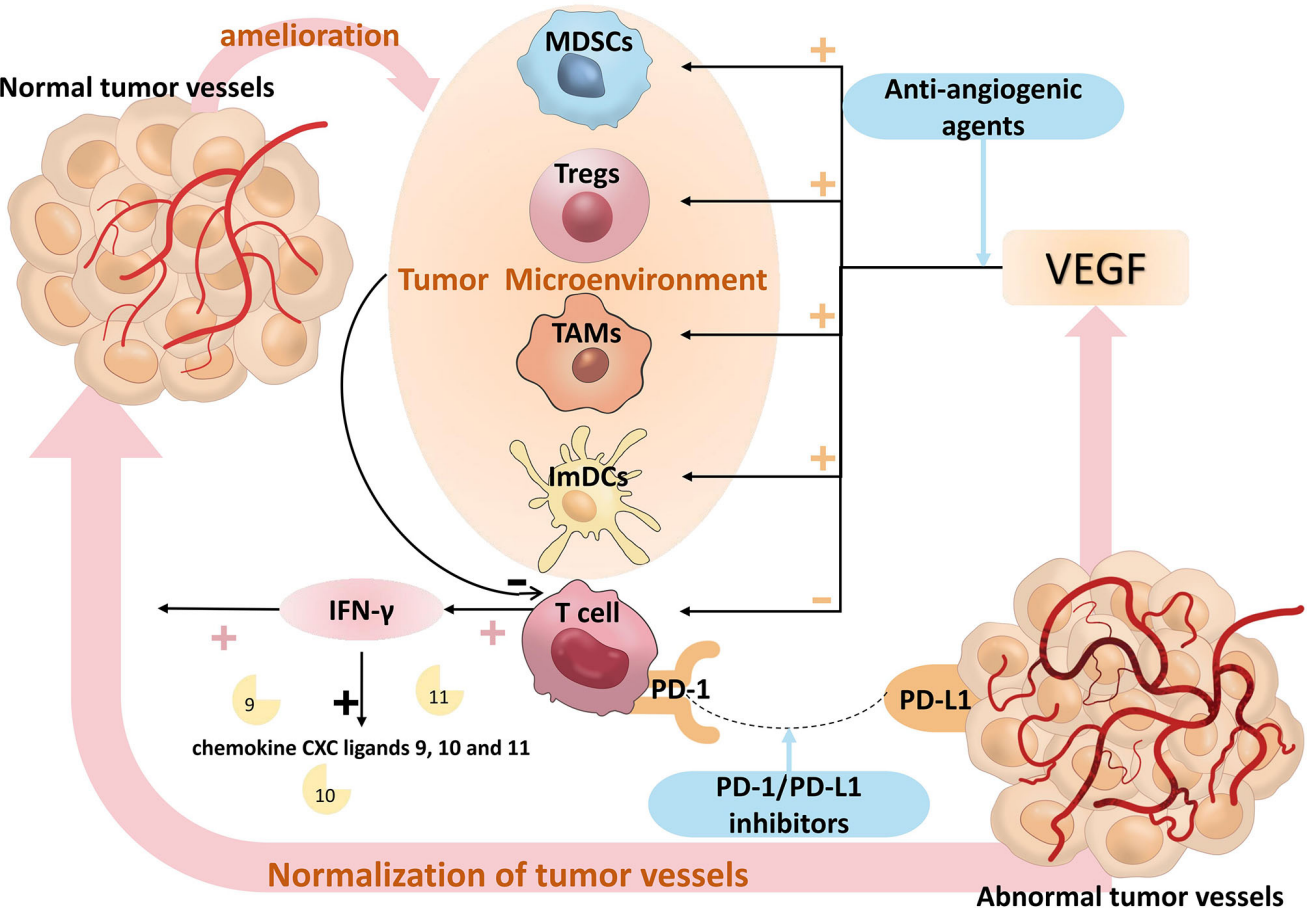
The relevant mechanisms of PD-1/PD-L1 inhibitors plus anti-angiogenic agents. T cells can be suppressed by members of the VEGF family, and VEGF can augment the suppressive actions of ImDCs, Tregs, TAMs, and MDSCs, which prevent T cells from having an anti-tumor impact. Anti-angiogenic agents inhibit the VEGF signaling pathway, which in turn activates T cells. T cells improve the tumor microenvironment by releasing IFN-γ, and IFN-γ upregulates chemokine CXC ligands 9, 10, and 11 to promote normalization of tumor vessels, thereby attenuating the effect of VEGF. PD-1/PD-L1 signaling pathway attenuates the anti-tumor activity of T cells, PD-1/PD-L1 inhibitors reactivate the anti-tumor activity of T cells, T cells improve the tumor microenvironment by releasing IFN-γ, IFN-γ upregulates chemokine CXC ligands 9, 10 and 11 to promote normalization of tumor vessels, thereby attenuating the effect of VEGF, which form a positive feedback loop between immunotherapy and anti-angiogenic therapy. VEGF, Vascular Endothelial Growth Factor; ImDCs, Immature Dendritic Cells; Treg, Regulatory T cells; TAMs, Tumor Associated Macrophages; MDSCs, Myeloid Derived Suppressor Cells. IFN-γ, Interferon-γ.

Activated immune cells can control tumor angiogenesis directly and indirectly at the same time. CD8+ T cells play a key role in inhibiting tumor angiogenesis through the secretion of Interferon-γ (IFN-γ). Endothelial cell proliferation is reduced and migration is diminished in response to IFN-γ, which promotes the secretion of IFN-Inducible Protein-10 (IP-10) and Monokine Induced by Interferon Gamma (MIG), which react with chemokine receptor 3 to inhibit endothelial cell proliferation and tumor angiogenesis (48, 49), thereby normalizing vasculature and promoting effector T cell infiltration. IFN-γ also downregulates VEGF-A and upregulates chemokine CXC ligands 9, 10 and 11, which together stimulate vascular maturation by enhancing the recruitment of pericytes (50). The IFN-γ/STAT1 signaling pathway promotes the reprogramming of M1-like TAMs and contributes to vascular normalization (51). CD4+ Th1 cells can also contribute to tumor vascular normalization through the production of IFN-γ in TME. In multiple mouse models, depletion of CD4+Th1 cells reduced pericyte coverage and increased aberrant tumor vessels, while activation of CD4+Th1 cells improved vascular normalization (52, 53). Immune cells can also directly influence the phenotype and function of tumor vessels through various cytokines, such as cytokines that inhibit tumor angiogenesis (interferon-α, interleukin-12, interleukin-18 or tumor necrosis factor) and chemokines (CXCL9, CXCL10 or CCL21) (54–56). One of the essential preconditions for immunological activation is anti-VEGF/VEGFR medication therapy, and strong evidence shows that immune cell activation furthers vascular normalization, creating a positive feedback loop between immunotherapy and anti-angiogenic therapy. **Figure 8** provides an illustration of the pertinent processes.

On data related to the use of PD-1/PD-L1 inhibitors plus anti-angiogenic agents for second or later-line treatment of advanced non-small cell lung cancer, a phase I clinical study evaluated the use of ramucirumab in combination with pembrolizumab in the later-line treatment of different malignancies and included 27 patients with NSCLC, showing an ORR of 30% (57). Zhou Na et al. published the results of a phase IB clinical study of camrelizumab in combination with anlotinib as a second or later-line treatment option for patients with advanced non-small cell lung cancer (58), which showed that camrelizumab in combination with anlotinib showed some effectiveness with an overall median PFS of 8.2 months and a median OS of 12.7 months for patients. Among the different anlotinib dose groups, the cohort group with anlotinib 12 mg demonstrated better efficacy and safety. The European Society for Medical Oncology (ESMO) 2021 reportd the results of the first interim analysis of the ORIENT-31 study (59) of sintilimab in combination with bevacizumab and chemotherapy in EGFR-mutated non-squamous NSCLC that has failed EGFR-TKI therapy, in the intention-to-treat population, patients in the sintilimab in combination with bevacizumab and chemotherapy arm achieved a significant prolongation of PFS compared to patients in the chemotherapy arm based on blinded independent imaging assessment committee assessment (median PFS: 6.9 months vs 4.3 months, HR=0.464, 95% CI: 0.337-0.639, p<0.0001).Wang Peiliang et al. conducted a retrospective analysis of the efficacy and safety of PD-1 inhibitors plus anlotinib in patients with advanced non-small cell lung cancer after failure of previous systemic therapy (60), which also showed anti-tumor activity and tolerable adverse effects of immune checkpoint inhibitors in combination with anti-angiogenic agents. Zhang Fan’s team and Hu Ran’s team also conducted a retrospective study on the efficacy and safety of immune checkpoint inhibitors in combination with chemotherapy and anti-angiogenic drugs as a second or later-line treatment option for advanced non-small cell lung cancer (61, 62), demonstrating the feasibility of immune checkpoint inhibitors in combination with chemotherapy and anti-angiogenic drugs in the second or later-line treatment. All of these findings provide some evidence-based evidence for the use of PD-1/PD-L1 inhibitors plus anti-angiogenic agents.

In conclusion, our study has a number of advantages: First and foremost, it was a real-world study that accurately reflected how PD-1/PD-L1 inhibitors combined with anti-angiogenic agents with or without chemotherapy would appear in the real world. Second, to show the connection between the research protocol and the risk of disease progression and death, the study applied rigorous statistical adjustments to reduce the impact of confounding factors. The study’s theoretical underpinnings were also well-established. A stratified analysis was then performed to confirm the consistency of our results within the subgroup. The study’s findings are valuable for physicians in that they may be used to create more effective treatment strategies for various individuals in a clinical environment.

Despite the significance of the study’s findings, there are a few restrictions on it. First of all, because the cases were gathered from a single center, the retrospective form of the research made it easy to add selection bias and skew the results of the relationships that were found. Second, the study’s limited sample size could have produced some insufficient statistical findings, and a sizable prospective clinical trial might be required to further support the study’s findings.

## Conclusion

PD-1/PD-L1 inhibitors plus anti-angiogenic agents with or without chemotherapy were superior to PD-1/PD-L1 inhibitors plus chemotherapy as second or later-line therapy in patients with advanced non-small cell lung cancer. This conclusion needs to be further validated in large-scale and prospective clinical trials.

## Data availability statement

The raw data supporting the conclusions of this article will be made available by the authors, without undue reservation.

## Ethics statement

The studies involving human participants were reviewed and approved by The Guangxi Medical University Affiliated Cancer Hospital ‘s ethical committee. The patients/participants provided their written informed consent to participate in this study.

## Author contributions

SC conducted the statistical analysis of the data and wrote the Chinese manuscript. HWe and WZ helped to perform the analysis and translated the article and created some of the graphics. WJ, RN, SZ, LT, HWa, CS, JH and AZ obtain informed consent from patients and administering treatment of patients and provide the clinical data of patients. YZ given some constructive discussions make revisions to the paper. QY conceptualized the research process and guided its conduct. All authors contributed to the article and approved the submitted version.
